# Kinetic Potentiometry as a Method for Studying the Interactions of Antioxidants with Peroxyl Radicals

**DOI:** 10.3390/antiox12081608

**Published:** 2023-08-14

**Authors:** Elena Gerasimova, Elena Salimgareeva, Dinara Magasumova, Alla Ivanova

**Affiliations:** Analytical Chemistry Department, Institute of Chemical Engineering, Ural Federal University, 19 Mira Str., 620002 Ekaterinburg, Russia; e.l.gerasimova@urfu.ru (E.G.); e.r.gazizullina@urfu.ru (E.S.); igdisanova.2011@mail.ru (D.M.)

**Keywords:** kinetic potentiometry, peroxyl radicals, inhibitor, rate constant, antiradical capacity

## Abstract

This work presents a new method using kinetic potentiometry to study the thermodynamic and kinetic parameters of the reactions of antioxidants with peroxyl radicals. The rate constants of the reaction of antioxidants with radicals have been determined, and the groups of “fast” and “slow” antioxidants have been conventionally distinguished. Fast antioxidants include ascorbic, uric, gallic, chlorogenic, caffeic acids, glutathione, L-cysteine, and catechol with constant values from (1.05–9.25) × 10^3^ M·s^−1^; “slow” antioxidants are α-tocopherol (in aqueous media), ionol, 2,6-ditretbutylphenol, and compounds of the azoloazine series, modified with polyphenolic fragments, with constant values from (4.00–8.50) × 10^2^ M·s^−1^. It is shown that the value of the rate constant is directly related to the type of kinetic dependence of the potential recorded when an antioxidant is introduced into the solution of the radical initiator. It is shown that the method with the determination of the induction period is difficult in the study of “slow” antioxidants. It has been established that the area above the curve of the kinetic dependence Exp(∆E) is directly related to the amount of inhibited peroxyl radicals and can be used to assess the inhibitory properties of an antioxidant from a thermodynamic point of view. “Fixed time method” and “Initial rate method” were used. Positive correlations between the described method have been established. The utility of the parameter of the area above the curve of the kinetic dependence Exp(∆E) in the study of objects of complex composition is shown.

## 1. Introduction

Reactive oxygen species (ROS), most of which are free radicals, play a significant role in the regulation of basic functions of cells, both under normal conditions and under the influence of various pathogenic factors on the cell [1]. ROS are not only by-products of chemical reactions but also participants in various cellular processes, namely, protection against pathogenic microorganisms, fertilization, cell division, apoptosis, regeneration, coordination of the direction of cell movement, regulation of vascular tone, etc. The balance between production and elimination of ROS results in intracellular homeostasis. Excess production of ROS can have a direct destructive effect on cellular structures, as well as initiate free radical oxidation of lipids, proteins, and nucleic acids, which underlies the pathogenesis of many diseases [1,2]. In this regard, the study of the inhibition processes of free radical oxidation by molecules with antiradical properties is very relevant. However, high reactivity limits the possibilities of using direct detection methods, the EPR and the chemiluminescent methods are the main ones [3,4]. The inhibition of free radical oxidation is more often studied using the oxidation processes of a model substrate [5,6].

The AAPH (2,2′-azobis-(2-amidinopropane) hydrochloride) is the most common radical generating system. Peroxyl radicals are formed during the thermal decomposition of AAPH [7,8,9,10,11]. The use of radical generating models is quite common in the study of compounds with antioxidant/inhibitory properties. This is due to the fact that, firstly, the generated peroxyl radicals model secondary peroxyl radicals formed in the body during the biomolecule oxidation. Secondly, the generation of radicals occurs in time at a certain rate, which is also similar to the radicals of the body, in contrast to model systems with stable radicals [7,8]. The fluorescent, the chemiluminescent, and the spectrophotometric methods are used to assess the inhibitory effect of antioxidants in radical generating systems [9,10,11,12,13,14]. A multi-stage chemical process is implemented in the chemiluminescent and the fluorescent assays, which, in addition to the main reaction of inhibition of free radical processes, includes activation–deactivation reactions of the compounds used [11,12,13,14]. This may distort the results regarding the inhibitory ability of antioxidants. In spectrophotometric methods, most often, a model oxidation substrate is used [5,12]. Accordingly, information about the antioxidant properties of the studied objects will be distorted by the features of interaction with oxidizable substrates. In addition, spectrophotometric methods are limited by the ranges of determined concentrations in accordance with the Beers–Lamberts Law and the difficulties in analyzing objects with an initial color.

The reaction between generated peroxyl radicals and inhibitors/antioxidants is based on the mechanism of hydrogen atom transfer (HAT-based assays) [8,9,10], which in aqueous media can be considered as proton transfer accompanied by electron transfer. In this regard, it is promising to use simple reliable electrochemical methods to study such systems, although their use for radical generating systems is practically not described in the literature. The electron transfer must be followed by the change of the redox potential of the system. This makes it promising to use, in particular, the potentiometry for recording the described processes. Previously, for the first time, we proposed the potentiometric method for studying the generation reaction inhibition of peroxyl radicals on the example of the thermal decomposition of the AAPH, and for determining the antiradical properties of inhibitors of various chemical nature [15]. The method is based on the regular change of the potential in the processes of peroxyl radicals generation and their inhibition. The determined value in the described method was the antiradical capacity (ARC), calculated from the induction period, as in classical methods [15,16]. This value is thermodynamic, that is, it characterizes the amount of peroxyl radicals scavenger antioxidant molecule. However, one of the significant factors affecting the inhibition of free radical oxidation by antioxidants in the body is the kinetic component of the inhibition process—the reaction rate of the antioxidant with radicals. Therefore, the existing methods for studying antioxidants are based either on determining the parameters of “antioxidant activity”, associated with the kinetics of a reaction between an antioxidant and the prooxidant or radical, it reduces or scavenge, or “antioxidant capacity”, measuring the thermodynamic conversion efficiency of an oxidant probe upon reaction with an antioxidant [17]. At the same time, knowledge of both the kinetic and thermodynamic parameters of the reaction of an antioxidant with an oxidant model is necessary to predict the action of an antioxidant in vivo. Information about the antioxidant capacity will allow one to estimate the number of radicals that can be inactivated by the antioxidant molecule in the case of using free radical models. Information about the reaction rate of an antioxidant with radicals will make it possible to estimate the action rate of an antioxidant. This, in turn, will provide information on whether the antioxidant is a “fast” acting molecule, that is, provide a fairly rapid therapeutic effect, or will be consumed more slowly and, accordingly, act for a longer time [18]. There are successful strategies for using combinations of “fast” and “slow” antioxidants to ameliorate oxidative stress during assisted reproduction, in the treatment of diabetes and other pathological conditions [19,20]. At the same time, the success is confirmed in both in vivo and kinetic experiments in vitro.

In this study, for the first time, we propose the method of the kinetic potentiometry for determining both the antioxidant capacity of the studied inhibitors and the rate constants of the reaction of generated peroxyl radicals with antioxidants.

## 2. Materials and Methods

### 2.1. Equipment and Reagents

Potentiometric measurements were carried out using an Expert-pH pH-meter (OOO Ekoniks-expert, Moscow, Russia) with the EMF measurement function and the RS-232 interface. A two-electrode electrochemical cell was used. The working electrode was the EPV-1 redox platinum electrode, and the reference electrode was the EVL-1M silver/silver chloride electrode (Ag/AgCl/3 mol/dm^3^ KCl) (Gomel ZIP, Gomel, Belarus). The studies were carried out in a thermostated cell at 37 °C using the LOIP LT-205a circulation thermostat (ZAO LOIP, Saint Petersburg, Russia).

The following reagents were used in the work: 2,2’-azobis(2-amidinopropane) dihydrochloride (AAPH), α-tocopherol, 2,6-ditretbutylphenol, ionol, uric acid, glutathione (Sigma-Aldrich, St. Louis, MO, USA); K_4_[Fe(CN)_6_], K_3_[Fe(CN)_6_], KH_2_PO_4_, Na_2_HPO_4_·12H_2_O (Reakhim, Moscow, Russia); ascorbic acid, L-cysteine, catechol, gallic acid, chlorogenic acid, caffeic acid (Panreac, Barcelona, Spain). All reagents were analytical grade. The studies were carried out in phosphate buffer solution (PS) pH = 7.4 KH_2_PO_4_/Na_2_HPO_4_·12H_2_O.

### 2.2. Objects of Research

**Figure 1:**
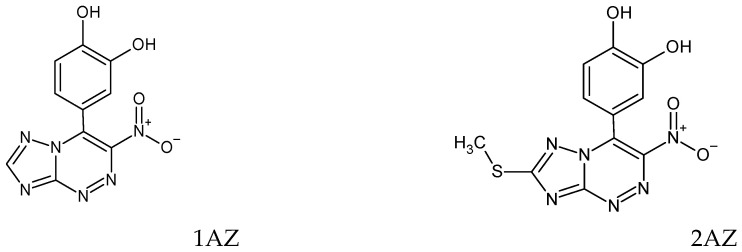
Chemical structures of substituted azoloazines.

**Figure 2:**
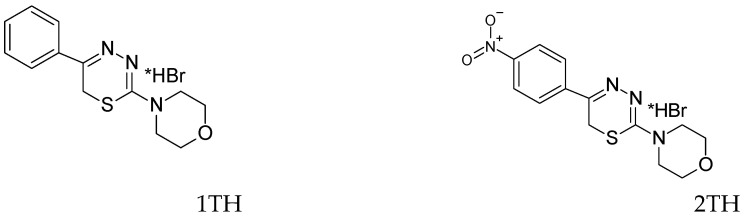
Chemical structures of substituted 1,3,4-6*H*-thiadiazines.

The objects of the research were solutions of antioxidants of natural and synthetic origin: ascorbic acid, uric acid, glutathione, cysteine, catechol, gallic acid, chlorogenic acid, caffeic acid, α-tocopherol, 2,6-ditretbutylphenol, 2,6-ditretbutyl-4-methylphenol (ionol), and compounds of the azoloazine series modified with polyphenol antioxidant fragments (Figure 1), as promising molecules for the creation of “double” action drugs [21]; derivatives of 6H-1,3,4-thiadiazines are newly synthesized compounds (Figure 2), promising antidiabetic drugs with pleiotropic activity [22].

### 2.3. Research Methods

#### 2.3.1. Determination of the Residual Concentration of Antioxidants

The residual concentration of antioxidants was determined by the potentiometric method using the K_3_[Fe(CN)_6_]/K_4_[Fe(CN)_6_] system [16,23]. The analytical signal is the change of the redox potential of the solution, measured between the working platinum electrode and the silver/silver chloride reference electrode when an antioxidant is added (1):

*q* [Fe(CN)_6_]3^−^ + AO → *q* [Fe(CN)_6_]^4−^ + AO_Ox_ (1)

where AO is the antioxidant; AO_Ox_ is the antioxidant oxidation product; *q* is the stoichiometric coefficient of the reaction between an antioxidant and potassium hexacyanoferrate (III).

The antioxidant concentration (M-eq) in the solution is calculated by the Formulas (2)–(3):

(2) $$C\left(AO\right)=\frac{C_{Ox}-\alpha C_{Red}}{1+\alpha }\cdot n$$

(3) $$\alpha =(C_{Ox}/C_{Red})\cdot 10^{\left(E_{2}-E_{1}\right)F/2.3RT}$$

where C_Ox_ is K_3_[Fe(CN)_6_] concentration, M; C_Red_ is K_4_[Fe(CN)_6_] concentration, M; *n* is sample dilution extent; E_1_ is initial potential of the system, V; E_2_ is potential, established in the system after the sample introduction, V; F is Faraday constant, C/mol; R is the gas constant, J/(mol∙K); T is the temperature, K.

To determine the residual concentration of model antioxidants during their interaction with generated peroxyl radicals, aliquots were taken from the reaction mixture at 37 °C at certain time intervals and placed in a solution containing the K_3_[Fe(CN)_6_]/K_4_[Fe(CN)_6_] system (at 25 °C).

The determination was carried out in the phosphate buffer solution (PBS) pH 7.4. The concentrations of K_3_[Fe(CN)_6_] and K_4_[Fe(CN)_6_] were 1 mM and 0.01 mM, respectively.

#### 2.3.2. Potentiometric Method for Determining Antiradical Capacity Using a Radical Generating System

The determination of the antiradical capacity (ARC) was carried out by the potentiometric method using 2,2′-azobis(2-amidinopropane) dihydrochloride (AAPH) as a model of the oxidant of the radical generating system [15,16]. The method is based on the direct interaction of thermally generated radicals with antioxidants in the solution. It consists in determining the regular change of the potential of the reaction mixture due to the reaction of radicals initiation and their inhibition by antioxidants. The determined parameter is the induction period (τ, s). ARC is calculated as the product of the generation rate of peroxyl radicals (W_i_, M·s^−1^) and the induction period, shown in Equation (4):

ARC = W_i_∙τ (4)

## 3. Results

The kinetic scheme of the interaction of an antioxidant with RO_2_^•^ radicals, formed during the decomposition of initiator I, includes the following reactions [13,14,15]:

1. Initiation:
  I → 2 RO_2_^•^ (5)
  Wi = 2*k_i_*·[I] (6)
2. Recombination/disproportionation of the RO_2_^•^:
  RO_2_^•^ + RO_2_^•^ → products (*k*_2_) (7)
3. Interaction with AO:
  RO_2_^•^ + AO_Red_ → RO_2_^−^ + AO_Ox_ (*k_inh_*) (8)
  where *k_i_* is the rate constant of peroxyl radicals generation of (1 × 10^6^ s^−1^); *k*_2_ is the rate constant of peroxyl radicals recombination (2.6 × 10^4^ s^−1^); *k_inh_* is the rate constant of the reaction of an antioxidant with peroxyl radicals.

**Figure 3:**
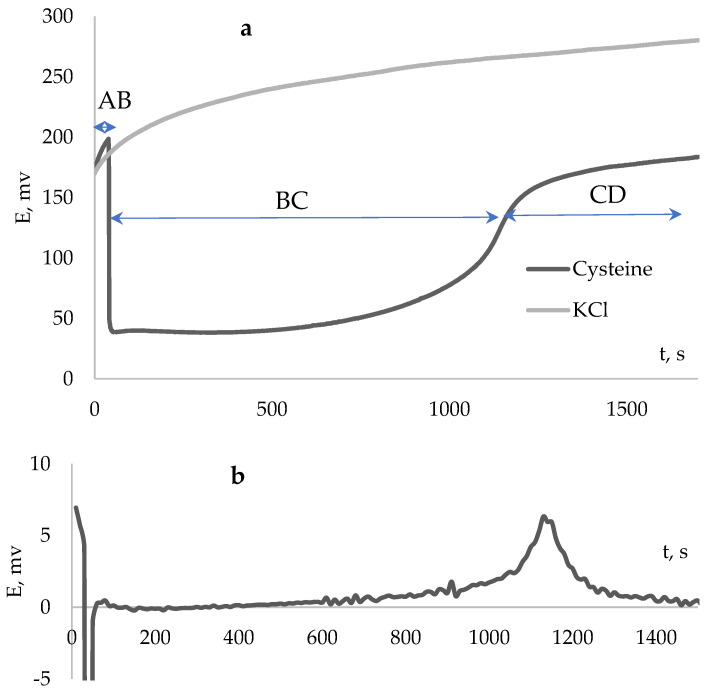
The kinetic curve of the potential change (**a**) and the derivative of the potential dependence on time (**b**) with the addition of cysteine (Cys) (C_Cys_ = 0.1 mM) to 0.1 M AAPH.

Previously, we established the regularity in the change of the redox potential of the system during the generation of peroxyl radicals and the reaction of antioxidant (inhibitors) with generated radicals [13,14]. The typical kinetic curve of potential change upon the addition of gallic acid to the AAPH solution is shown in Figure 3.

Area AB corresponds to the initiation of a radical reaction, BC to the interaction of generated radicals with an inhibitor (induction period), CD to the end of the induction period and further radical initiation. It should be noted that when a compound that does not have inhibitory properties is introduced into the initiator solution, for example, potassium chloride, the potential change will correspond to a continuous increase, that is, initiating a radical reaction.

The kinetic dependences of the potential are similar to the curves of chemiluminescent glow in the presence of activators obtained by introducing an inhibitor into an initiator solution [13,14]. The completion of the induction period indicates the depletion of antioxidants in the system and is accompanied by a sharp increase of the potential, which, in this case, can be determined at the inflection point of the kinetic dependence of the potential (the maximum of the first derivative (dE/dt)max, Figure 3a). The effectiveness of an inhibitor using thermodynamic parameters, such as antiradical capacity and inhibition coefficient, can be estimated based on the induction period [15,16]. Determining the thermodynamic parameters of the interaction of antioxidants with free radicals is the most common method for studying antioxidant properties [9,10,11,12,14,15], both for stable radicals and for radical generating systems. However, traditionally, to study the action of biologically active compounds, both methods for studying pharmacodynamics and pharmacokinetics are used [18,24]. Methods to the determination of the kinetic parameters of the reaction of antioxidants with free radicals are limited in the world literature studies of antioxidants [12,13]. At the same time, this information is also of key importance, as well as information on the amount of absorbed radicals by the antioxidant molecule. Chemiluminescent methods are described that make it possible to evaluate the thermodynamic and kinetic parameters of the reaction of the antioxidants with free radicals. However, a multistage chemical process is implemented in these methods, which includes activation–deactivation reactions of luminol, in addition to the main reaction of the inhibition of free radical processes [12,13,14]. This stage has its own kinetics, depending on the rate of reaction with the activator and the concentrations ratio of the antioxidant and the generated radicals. In this regard, the information is difficult to interpret in relation to the inhibitory ability of antioxidants. While the potentiometric method is devoid of these disadvantages. It is simple, express, economical, and present in almost any laboratory, even with limited equipment.

Based on the previously described relationships [15,16] between potential changes in the reactions of initiation and inhibition, in the framework of this study, a new method was proposed that makes it possible to use the method of kinetic potentiometry to study antioxidant properties, taking into account the kinetic features of the antioxidants reaction with free radicals. Despite the fact that traditionally the method of kinetic potentiometry has very narrow limits of applicability, in most cases based on the use of potentiometric sensors [25,26], the present studies show its broader possibilities. The use of the kinetic method makes it possible to conduct studies under dynamic conditions when the concentrations of antioxidants and radicals change with time.

**Figure 4:**
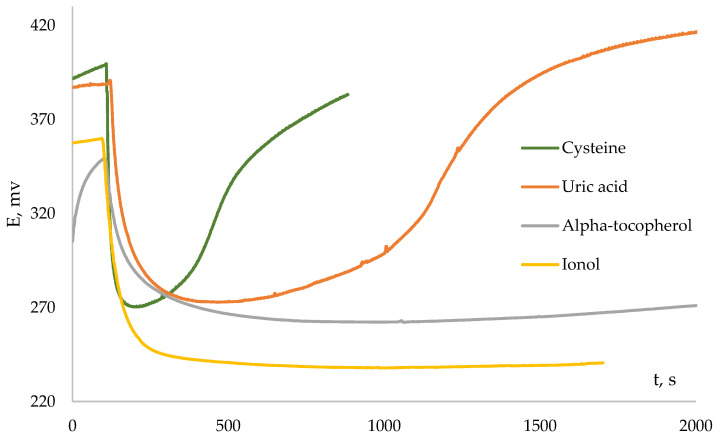
The kinetic curve of the potential changes with the addition of antioxidant (C_AO_ = 0.1 mM) to 0.1 M AAPH.

In the previously described system of interaction of generated peroxyl radicals with an inhibitor, it is obvious that the reaction regularities will largely depend on the ratio of the rates of initiation (*k_i_*) and inhibition (*k_inh_*). Figure 4 shows the kinetic dependences of the potential for some antioxidants.

A number of antioxidants are characterized by the change of potential, described above, with a pronounced inflection point, as can be seen from the figure. Such antioxidants, according to the conducted potentiometric studies, include ascorbic and uric acids, cysteine, glutathione, compounds of the polyphenol series: catechol, gallic, chlorogenic, and caffeic acids, that is, the most common endogenous and exogenous antioxidants. In addition, according to the literature data, these antioxidants are characterized by rather high values of the reaction rate constants with various ROS [25] determined using the EPR method.

**Figure 5:**
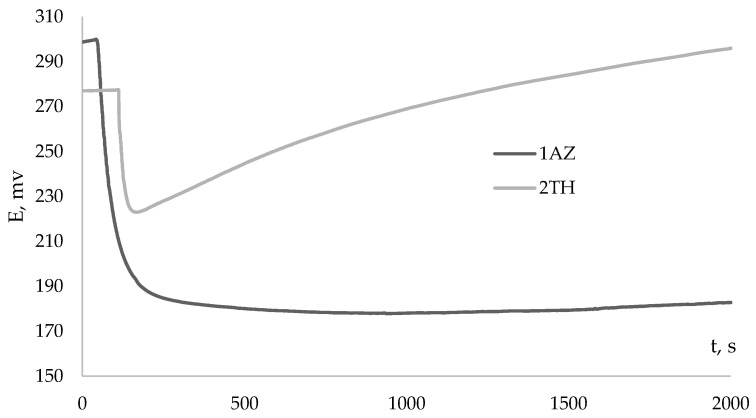
The kinetic curve of the potential changes with the addition of 1AZ и 1TH (C_AO_ = 0.1 mM) to 0.1 M AAPH.

However, for some antioxidants, the kinetic dependences of the potential on time, when an antioxidant is introduced into the AAPH solution, do not have a pronounced inflection point and, accordingly, do not obey the described classical patterns [15]. Dependencies of this type were obtained for lipophilic α-tocopherol in an aqueous medium and synthetic sterically hindered phenols—ionol (2,6-ditretbutyl-4-methylphenol) and 2,6-ditretbutylphenol. The patterns are consistent with the literature data on the relatively low rates of the reaction of α-tocopherol with free radicals in aqueous media and the structural features of the interaction of sterically hindered phenols with radicals [26,27]. In addition, dependences of this type were registered for synthesized compounds used in the treatment of various diseases: derivatives of 6H-1,3,4-thiadiazines—newly synthesized compounds, promising antidiabetic drugs with pleiotropic activity (antioxidant and antiglycation) [22], which have a prolonged activity, and for compounds of the azoloazine series modified with polyphenolic antioxidant fragments that exhibit antiviral and antioxidant activity [21]. Figure 5 shows the kinetic dependences of the potential for thiadiazine (1AZ) and azoloazine (1TH) derivatives.

Thus, information about the values of the rate constants of the reaction of the studied inhibitors with peroxyl radicals under the experimental conditions is necessary to determine the relationship between the type of kinetic dependence of the potential when the inhibitor is introduced into the initiator solution.

### Determination of the Reaction Rate Constant of an Antioxidant with Peroxyl Radicals

In this work, the new potentiometric method for determining the rate constant of the reaction of an antioxidant with generated peroxyl radicals was proposed. For this purpose, during the interaction of an antioxidant with AAPH radicals, aliquots were taken from the reaction mixture at certain time intervals, and the residual concentration of the antioxidant was determined by the potentiometric method using the K_3_[Fe(CN)_6_]/K_4_[Fe(CN)_6_] system [23].

The contribution of the radical recombination reaction (*k*_2_) increases with a decrease in the residual concentration of the antioxidant as the induction period approaches completion. In this situation, the reaction contribution of the generated radicals with K_4_[Fe(CN)_6_] (8) can be neglected, and it can be assumed that the potential change is due only to an increase in the concentration of K_4_[Fe(CN)_6_], which occurs in the reaction with the antioxidant (1).

RO_2_^•^ + [Fe(CN)_6_]^4−^ → RO_2_^−^ + [Fe(CN)_6_]^3−^ (9)

Thus, the residual concentration of the antioxidant was estimated during the entire induction period by the example of the interaction of 0.05 mM, 0.1 mM, 0.2 mM solutions of antioxidants (ascorbic acid, glutathione, cysteine, polyphenolic compounds, α-tocopherol, ionol, 2,6-ditretbutylphenol, compounds of the azoloazine series) with 0.1 M AAPH solution at 37 °C.

**Figure 6:**
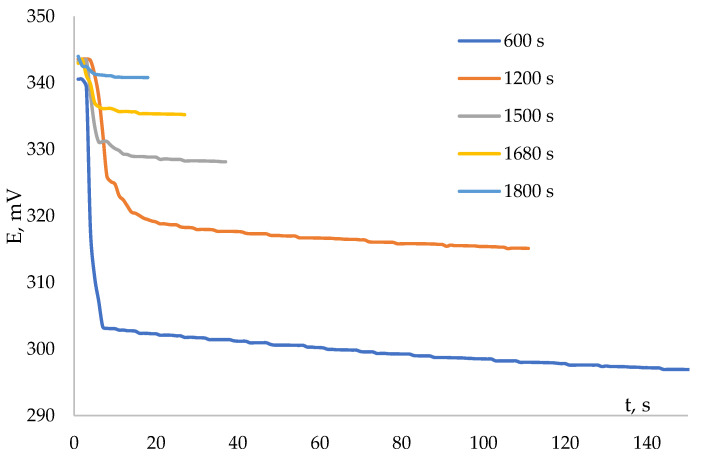
Kinetic dependences of the potential in the interaction of the K_3_[Fe(CN)_6_] (0.01 M)/K_3_[Fe(CN)_6_] (0.1 mM) system with the 0.2 mM solution of ascorbic acid from the reaction mixture with 0.1 M AAPH and different sampling times.

Figure 6 shows the kinetic dependences of the potential on time with the addition of an aliquot from the reaction mixture (interaction of the antioxidant with the AAPH) into the solution containing K_3_[Fe(CN)_6_]/K_4_[Fe(CN)_6_] in a ratio of 0.01 M/0.1 mM.

**Figure 7:**
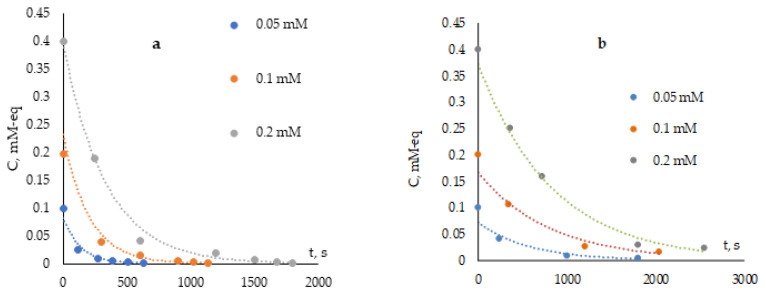
Dependence of the residual concentration of ascorbic acid (**a**) and α-tocopherol (**b**) on the time of interaction with 0.1 M AAPH solution.

Figure 7a,b show dependences of the residual concentration of antioxidants on the time of interaction with the AAPH.

The reaction of the generated radicals with an antioxidant (8) is a second-order reaction (10). However, taking into account that the equilibrium concentration of the generated radicals [RO_2_^•^] is incommensurably low compared to the inhibitor concentration [InH] (11), the reaction can be taken as a pseudo-first-order reaction. The rate of this reaction (interaction of the antioxidant with peroxyl radicals) (8) is limited by the generation rate of peroxyl radicals. Therefore, it is difficult to apply the classical approach to calculating the first-order reaction constant for this system.

W = *k_inh_* [RO_2_^•^] [InH] (10)

[RO_2_^•^] << [InH] (11)

Therefore, the method, based on determining the initial slope of the semilogarithmic anamorphoses of the kinetic curves of the change in concentration on time in conditions of low acceptor concentrations, was used [28]. In this case, the consumption rate of the inhibitor will be described by the expression for the rate of the first-order reaction (12), where the coefficient *a* will be equal to expression (13). In these conditions, the consumption rate of the acceptor will tend to expression (14), and the limiting value at an acceptor concentration tending to zero can be determined graphically. The value of *k_inh_* (15) can be estimated using the value of the recombination constant [28] equal to 2*k*_2_ = 5 × 10^4^ (M·s^−1^).

ln [InH] = ln [InH]_0_—*a*·t (12)

*a* = *k_inh_*·[RO_2_^•^] (13)

at [InH] → 0  *a* → *k_inh_*·(W_i_/2*k*_2_)^0.5^ (14)

*k_inh_* = [dln(InH)/dt]_C(InH)→0_/(W_i_/2*k*_2_)^0.5^ (15)

**Figure 8:**
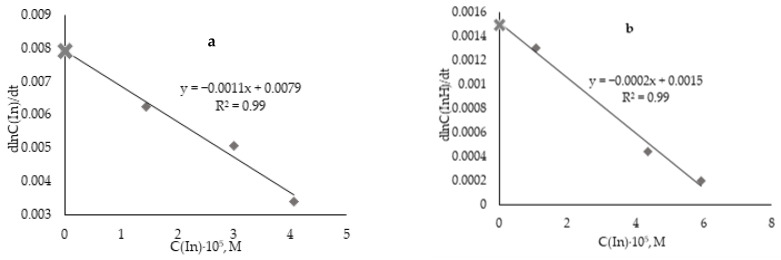
Dependence of the initial slope of semilogarithmic anamorphoses (dlnC(In)/dt) of kinetics curves on the initial concentration of ascorbic acid (**a**) and α-tocopherol (**b**).

Figure 8a,b show dependences of the initial slope of the semilogarithmic anamorphoses of the kinetic curves on the initial concentration of the inhibitor—ascorbic acid and α-tocopherol, respectively.

The *k_inh_* values of the studied antioxidants were calculated as described above, and the results are presented in Table 1.

According to the rate constants of the reaction with peroxyl radicals, antioxidants can be conditionally divided into “fast” and “slow”. “Fast” antioxidants include ascorbic, uric, gallic, chlorogenic, caffeic acids, glutathione, L-cysteine, and catechol with constant values from (1.05–9.25) × 10^3^ M·s^−1^. “Slow” antioxidants include α-tocopherol (in aqueous media), ionol, 2,6-ditretbutylphenol, and compounds of the azoloazine series 1AZ and 2AZ with constant values from (4.00 to 8.50) × 10^2^ M·s^−1^. These trends in the differences in the rate constants of antioxidants were also noted by other researchers [29,30]. However, it is not possible to compare absolute values due to variations in experimental conditions.

It is not possible to calculate the constants of interaction with peroxyl radicals by the proposed method for compounds of the thiadiazine series. Thiadiazines are capable of transformation into substituted mercaptopyrazoles [31], which react with potassium hexacyanoferrate (III). At the same time, the rate of this transformation is low and, accordingly, the rate of the reaction with potassium hexacyanoferrate (III) is low for the thiadiazine derivatives [22]. This does not allow the correct determination of the residual concentration of thiadiazines in conditions of competing kinetics: the initiation of a radical reaction and the reaction of thiadiazines in potassium hexacyanoferrate (III).

Another potentiometric method to the study of antiradical properties, which makes it possible to study both “fast” and “slow” antioxidants, including antioxidants with specific transformation kinetics, was proposed to determine the thermodynamic parameters of such compounds.

Development of the method of the potentiometric study of antiradical properties in view of the kinetic features of the interaction of antioxidants with peroxyl radicals.

The classical chemiluminescent methods, described in the literature [12,30], have different ways of assessing the inhibitory properties. One such method is to calculate the area under the luminescence intensity curve, since it is directly proportional to the concentration of uninhibited radicals.

By an analogy with the classical kinetic methods [32,33], “Fixed time method” and “Initial rate method” can be used in the potentiometry. The absolute value of the potential cannot be selected as a changeable parameter in the “Initial rate method” because there is no potential pair and the equilibrium of the system is not achieved in the described method. In this regard, the method has been proposed in which the achievement of the initial value by the potential before the inhibitor introduction can be used as a determining parameter. The measured potential is directly proportional to the logarithm of the concentration of generated peroxyl radicals~ln[RO_2_^•^], and therefore, the potential difference (∆E) will be directly proportional to the logarithm of the change in the concentration of radicals ~ln[∆RO_2_^•^]. Thus, the area above the curve of the kinetic dependence Exp(∆E), when reaching 1, is directly related to the amount of inhibited peroxyl radicals and characterizes the inhibitory properties of the antioxidant from a thermodynamic point of view.

**Figure 9:**
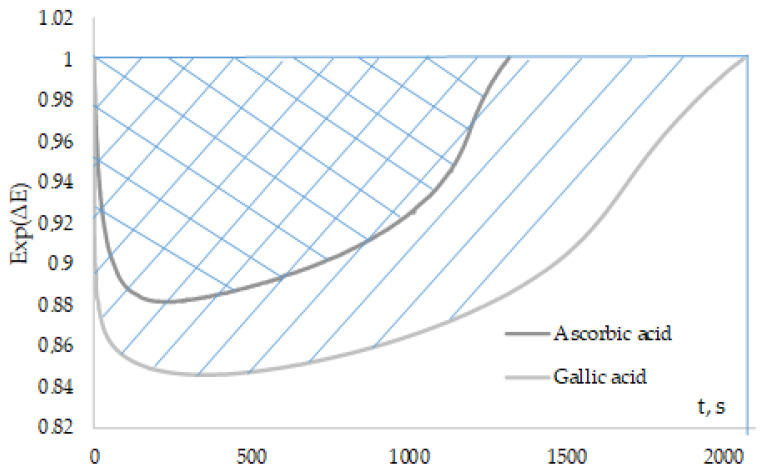
Kinetic dependences of Exp(∆E) with the added 0.1 mM ascorbic and gallic acids to 0.1 M AAPH.

The areas above the curves of the kinetic dependences Exp(∆E) of “fast” antioxidants were calculated using the proposed method (Figure 9).

The calculation of the area is limited by the time, which, in this case, is selected by the value of the potential corresponding to the introduction of the inhibitor, that is, until Exp(∆E) reaches unity.

“Fixed time method” is possible to use. At the same time, the reasonable time for analysis of 1200 s was chosen to calculate the area by analogy with chemiluminescent approaches [12,32]. In addition, the optimal time for monitoring the indicator reaction rate is 5–15 min in classical kinetic methods. The selected time corresponds to the middle of this interval.

The areas of “fast” antioxidants, calculated in different methods, are shown in Table 2.

It can be noted from the obtained values that gallic acid, catechol, and caffeic acid have the most pronounced inhibitory properties.

The correlation degree between the method for determining the induction period and the “Initial rate method” and “Fixed time method” was 0.86 and 0.87, respectively (r_crit_ = 0.75, *n* = 7, *p* = 0.95). The positive correlation of areas with antiradical capacity indicates the possibility of using this method in the study of antiradical properties.

The “Initial rate method” is difficult to use for “slow” antioxidants because reaching Exp(∆E) unity can take a long time. Therefore, the “Fixed time method” was used for this type of antioxidants.

**Figure 10:**
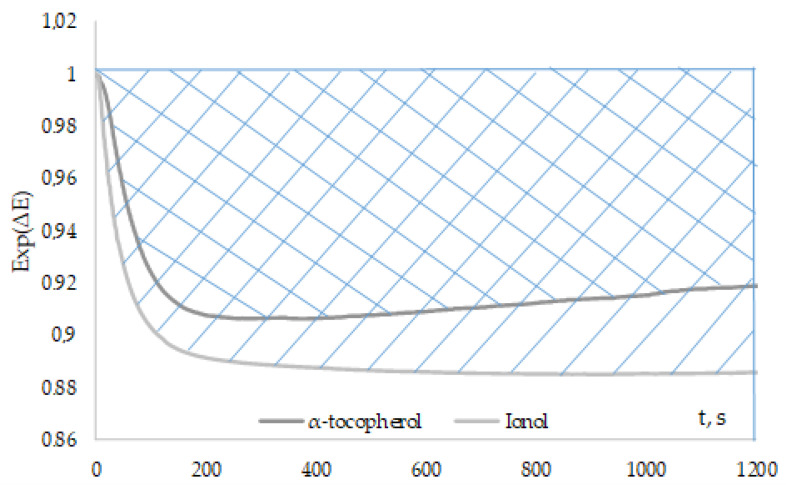
Kinetic dependences of Exp(∆E) with the addition of 0.1 mM α-tocopherol and ionol to 0.1 M AAPH.

The areas above the curves of the Exp(∆E) kinetic dependences of “slow” antioxidants were calculated using the proposed method (Figure 10).

The areas of “slow” antioxidants are shown in Table 3.

It can be noted that ionol and 2,6-ditretbutylphenol have the most pronounced inhibitory properties, and the areas are 130 and 189, respectively.

**Figure 11:**
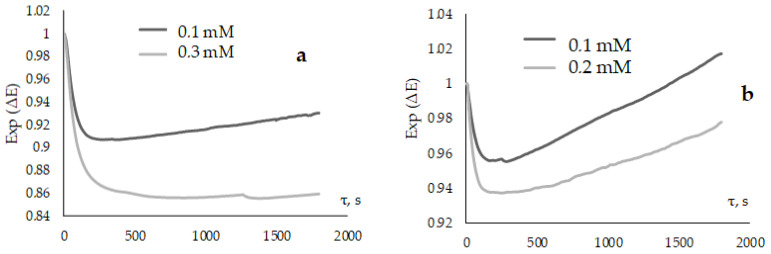
Exp(∆E) kinetic dependences with the addition of α-tocopherol (**a**) and 2AZ (**b**) at various concentrations to 0.1 M AAPH.

Relationships between the area over the kinetic dependence of the potential on the concentration of the “slow” antioxidant were studied to confirm the validity of using the proposed method. Figure 11a,b shows the Exp(∆E) kinetic curves for the α-tocopherol and 2AZ azoloazine derivative. Table 4 shows the areas for different concentrations of these compounds. 

It can be seen from the values of Table 3 that the higher the antioxidant concentration, the greater the value of the inhibitory ability, estimated by the proposed method. This again confirms the possibility of using the method.

Since real objects of complex composition, for example, of plant origin, in most cases contain a mixture of antioxidants with different kinetic characteristics, mixtures of “fast” and “slow” antioxidants were studied, for example, the mixture of ascorbic acid and α-tocopherol at various concentrations.

**Figure 12:**
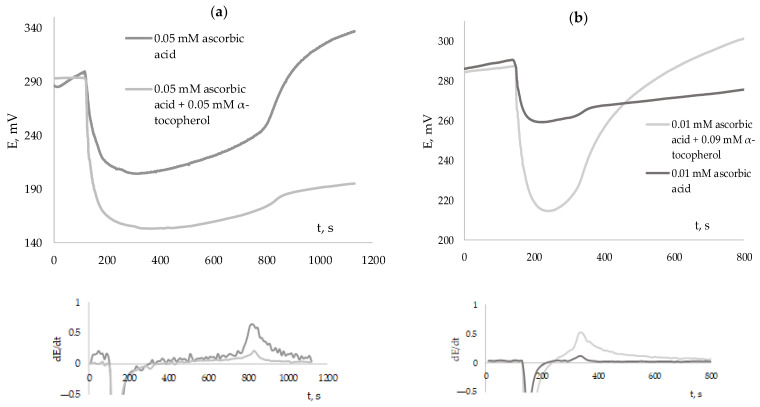
The kinetic curve of the potential change and the derivative of the potential dependence on time with the addition of ascorbic acid and ascorbic acid mixed with α-tocopherol in various ratios to the solution (0.05 mM ascorbic acid and 0.05 ascorbic acid + 0.05 mM α-tocopherol (**a**); 0.01 mM ascorbic acid and 0.01 ascorbic acid + 0.09 mM α-tocopherol (**b**)).

Figure 12 shows the kinetic dependences of the potential on the addition of ascorbic acid and ascorbic acid mixed with α-tocopherol in various ratios to the solution.

As can be seen from the presented figures, if the object is the mixture of “fast” and “slow” antioxidants, then the induction period and, accordingly, the antiradical capacity will be determined by the presence of a “fast” antioxidant in the mixture. The presence of a “slow” antioxidant in the mixture does not affect the position of the inflection point of the first derivative of the potential on time. ARC of the 0.075 mM solution of the ascorbic acid and the mixture of the 0.075 mM ascorbic acid and 0.075 mM α-tocopherol will be equal to (0.146 ± 0.008) mM-eq and (0.147 ± 0.006) mM-eq, respectively. However, significant differences are observed when calculating the area over the kinetic dependence Exp(∆E) by the “Fixed time method”. The area of the solution of individual ascorbic acid is (82 ± 6) s, the solution of the equimolar mixture of ascorbic acid and α-tocopherol is (155 ± 11) s. At the same time, as can be seen in Figure 12b, the position of the inflection point remains almost unchanged even when the amount of the “slow” antioxidant is significantly exceeded. The area over the kinetic dependence Exp(∆E) by the “Fixed time method” of the solution of individual ascorbic acid is (9 ± 1) s, and the mixture of ascorbic acid (0.01 mM) and α-tocopherol (0.09 mM) is (95 ± 9) s.

Therefore, individual antioxidants of natural origin and extracts of plant origin, the antioxidant composition of which is determined by the content, mainly, of “fast” (polyphenolic) compounds, can be studied by the approach of determining the induction period. However, it is more expedient to use the approach of determining the area over the kinetic dependence Exp(∆E) for objects of synthetic origin and objects of complex composition containing mixtures of hydro- and lipophilic antioxidants.

## 4. Conclusions

For the first time, the approach using the method of kinetic potentiometry to determine the thermodynamic and kinetic parameters of the reaction of generated peroxyl radicals with antioxidants was proposed. The rate constants of the reaction of peroxyl radicals with inhibitors were determined by the proposed approach under conditions of low inhibitor concentrations and by using the system of potassium hexacyanoferrates. Antioxidants can be conventionally divided into “fast” and “slow” groups according to the rate constants. Information about the kinetic group of antioxidants can be important when they are used, for example, as a therapy. If you need a quick effect, then it is advisable to use “fast” antioxidants. But this effect will not be longer lasting, as there will be a rapid consumption of the substance. If it is advisable to obtain a less pronounced but prolonged effect, then it is reasonable to use “slow” antioxidants.

It has been reliably shown that the form of the kinetic dependence of the potential, recorded upon the introduction of an antioxidant into a solution of a radical initiator, will depend on the reaction rate of antioxidants with radicals. This fact will directly affect the determination of the thermodynamic parameters of the interaction of inhibitors with radicals. It has been established that the approach of determining the induction period is difficult for the analysis of “slow” antioxidants. Therefore, it was proposed to use the area parameter above the curve of the kinetic dependence Exp(∆E) because it is directly related to the amount of inhibited peroxyl radicals and characterizes the inhibitory properties of an antioxidant from a thermodynamic point of view. The kinetic methods of “Fixed time method” and “Initial rate method” were used. A positive correlation between the methods has been shown. It has been established that it is unreliable to use the induction period parameter in the study of objects of a complex composition containing a mixture of “fast” and “slow” antioxidants, and it is advisable to use the area above the curve of the kinetic dependence Exp(∆E). It should be noted that this is a completely new analytical application of the kinetic potentiometry method for studying radical inhibition reactions.

## Figures and Tables

**Table 1:** Rate constants of the reaction of antioxidants with peroxyl radicals (*k_inh_*) (T = 37 °C, pH = 7.4).

Antioxidant	*k_inh_,* 10^3^ s^−1^
Ascorbic acid	3.95 ± 0.12
Glutathione	9.25 ± 0.14
Uric acid	3.30 ± 0.10
L-cysteine	5.75 ± 0.09
Catechol	1.20 ± 0.02
Gallic acid	2.50 ± 0.03
Chlorogenic acid	1.05 ± 0.05
Caffeic acid	2.60 ± 0.08
α-tocopherol	0.75 ± 0.02
2.6-ditertbutylphenol	0.40 ± 0.02
Ionol	0.70 ± 0.03
1AZ	0.75 ± 0.01
2AZ	0.85 ± 0.02

**Table 2:** The area over the kinetic dependence Exp(∆E) for “fast” antioxidants (*n* = 5, *p* = 0.95, C(AAPH) = 0.1 M).

Antioxidant	S*_ARC_, s	S_r_	S**_ARC_, s	S_r_
Ascorbic acid	117 ± 9	0.10	98 ± 8	0.09
Uric acid	194 ± 18	0.11	162 ± 16	0.10
Glutathione	40 ± 4	0.12	36 ± 4	0.13
Catechol	287 ± 26	0.09	267 ± 28	0.11
Gallic acid	232 ± 21	0.10	202 ± 11	0.06
Chlorogenic acid	218 ± 22	0.12	201 ± 20	0.11
Caffeic acid	340 ± 33	0.11	333 ± 23	0.08

S*_ARC_—Initial rate method. S**_ARC_—Fixed time method.

**Table 3:** The area over the kinetic dependence Exp(∆E) for “slow” antioxidants (*n* = 5, *p* = 0.95, C(AAPH) = 0.1 M).

Antioxidant	S**_ARC_, c	S_r_
α-tocopherol	101 ± 9	0.10
2.6-ditrebutylphenol	189 ± 16	0.12
Ionol	130 ± 12	0.09
1AZ	30 ± 3	0.11
2AZ	36 ± 3	0.12

S**_ARC_—Fixed time method.

**Table 4:** The area over the Exp(∆E) kinetic dependence for some “slow” antioxidants (*n* = 5, *p* = 0.95, C(AAPH) = 0.1 M).

Antioxidant	C_AO_, mM	S**_ARC_, s	S_r_
α-tocopherol	0.1	101 ± 9	0.10
	0.3	258 ± 24	0.11
Ionol	0.05	92 ± 8	0.09
	0.1	130 ± 11	0.09
2AZ	0.1	36 ± 4	0.12
	0.2	65 ± 7	0.11

S**_ARC_—Fixed time method.

## Data Availability

All data generated or analyzed during this study are included in this article.
